# Effect of 3′UTR *RET* Variants on *RET* mRNA Secondary Structure and Disease Presentation in Medullary Thyroid Carcinoma

**DOI:** 10.1371/journal.pone.0147840

**Published:** 2016-02-01

**Authors:** Lucieli Ceolin, Mirian Romitti, Débora Rodrigues Siqueira, Carla Vaz Ferreira, Jessica Oliboni Scapineli, Beatriz Assis-Brazil, Rodolfo Vieira Maximiano, Tauanne Dias Amarante, Miriam Celi de Souza Nunes, Gerald Weber, Ana Luiza Maia

**Affiliations:** 1 Thyroid Section, Endocrine Division, Hospital de Clínicas de Porto Alegre, Universidade Federal do Rio Grande do Sul, Porto Alegre, RS, Brasil; 2 Pathology Department, Hospital de Clínicas de Porto Alegre, Universidade Federal do Rio Grande do Sul, Porto Alegre, RS, Brazil; 3 Department of Physics, Computational Biophysics Group, Universidade Federal de Minas Gerais, Belo Horizonte, MG, Brazil; National Cheng Kung University Medical College, TAIWAN

## Abstract

**Background:**

The *RET* S836S variant has been associated with early onset and increased risk for metastatic disease in medullary thyroid carcinoma (MTC). However, the mechanism by which this variant modulates MTC pathogenesis is still open to discuss. Of interest, strong linkage disequilibrium (LD) between *RET* S836S and 3'UTR variants has been reported in Hirschsprung's disease patients.

**Objective:**

To evaluate the frequency of the *RET* 3’UTR variants (rs76759170 and rs3026785) in MTC patients and to determine whether these variants are in LD with S836S polymorphism.

**Methods:**

Our sample comprised 152 patients with sporadic MTC. The *RET* S836S and 3’UTR (rs76759170 and rs3026785) variants were genotyped using Custom TaqMan Genotyping Assays. Haplotypes were inferred using the phase 2.1 program. *RET* mRNA structure was assessed by Vienna Package.

**Results:**

The mean age of MTC diagnosis was 48.5±15.5 years and 57.9% were women. The minor allele frequencies of *RET* polymorphisms were as follows: S836S, 5.6%; rs76759170, 5.6%; rs3026785, 6.2%. We observed a strong LD among S836S and 3’UTR variants (|D’| = -1, r^2^ = 1 and |D’| = -1, r^2^ = 0,967). Patients harboring the S836S/3’UTR variants presented a higher percentage of lymph node and distant metastasis (P = 0.013 and P<0.001, respectively). Accordingly, RNA folding analyses demonstrated different RNA secondary structure predictions for WT(TCCGT), S836S(TTCGT) or 3’UTR(GTCAC) haplotypes. The S836S/3’UTR haplotype presented a greater number of double helices sections and lower levels of minimal free energy when compared to the wild-type haplotype, suggesting that these variants provides the most thermodynamically stable mRNA structure, which may have functional consequences on the rate of mRNA degradation.

**Conclusion:**

The *RET* S836S polymorphism is in LD with 3’UTR variants. *In silico* analysis indicate that the 3’UTR variants may affect the secondary structure of *RET mRNA*, suggesting that these variants might play a role in posttranscriptional control of the *RET* transcripts.

## Introduction

Medullary thyroid carcinoma (MTC), a malignant tumor originating in parafollicular C cells of the thyroid, represents about 4% of all thyroid cancers [1]. MTC is mainly sporadic (75%), but may also be part of an inherited disorder transmitted as an autosomal dominant trait with 100% penetrance, referred to as multiple endocrine neoplasia type 2 (MEN 2). The MEN 2 syndrome is classified into three distinct clinical subtypes: MEN type 2A (MEN 2A), characterized by the presence of MTC, pheochromocytoma (PHEO), and hyperparathyroidism (HPT); MEN type 2B (MEN 2B), that includes MTC, PHEO, ganglioneuromatosis, and marfanoid habitus; familial MTC (FMTC), characterized by MTC as the only feature of the disease [2,3].

The *RET* (REarranged during Transfection) proto-oncogene is the susceptibility gene for hereditary MTC and *RET* molecular analysis is now considered critical in MTC management [4,5]. Genotype–phenotype correlation has been reported and mutations in exons 5, 8, 10, 11, 14, 15 or 16 occur in more than 98% of MEN2 families [4,5]. The RET gene, a member of the cadherin superfamily, encodes a receptor tyrosine kinases, which are cell-surface molecules that transduce signals for cell growth and differentiation. This gene plays a crucial role in neural crest development, and it can undergo oncogenic activation *in vivo* and *in vitro* by cytogenetic rearrangement. Alternative splicing of the *RET* gene results in the production of *RET51*, *RET43* and *RET9* isoforms, contain 51, 43 and 9 amino acids in their C-terminal tail, respectively. The biological roles of isoforms RET51 and RET9 are the most well studied *in-vivo* as these are the most abundant isoforms in which *RET* occurs [6]. RET9 and RET51 isoforms appear to be coexpressed in MTC [7,8]. The RET 51 isoform with MEN2-related mutations might activate the Ras signaling cascade with a greater efficiency than the RET 9 isoform. Nevertheless, it has not been observed significant differences in the transforming ability of RET51 and RET9 [6].

Although much is known about hereditary MTC, the molecular mechanisms underlying sporadic MTC tumors remain to be clarified. Somatic RET point mutations in exon 16 (M918T) and gene deletions are described in 23–66% of sporadic MTC [9–11]. However, these mutations are not uniform among the various cell subpopulations, suggesting that the tumor may be of polyclonal origin or that the RET mutations are not the initial events in MTC tumorigenesis. Thus, in the last years, several studies have also investigated the relationship between the presence of single nucleotide polymorphisms (SNPs) in the *RET* gene and susceptibility or progression of sporadic MTC. The *RET* neutral variants L769L, S836S, and S904S have been associated with clinical presentation and disease outcome in sporadic MTC patients [12]. Particularly, the S836S variant (codon 836 of exon 14, SerAGC/SerAGT) has been found over-represented in sporadic MTC patients from Germany, Spain, and the United States [13,14]. This variant also has been associated with two-to-three-fold increase in the risk of MTC in the Spanish population [14], early onset of MTC and increased risk for metastatic disease [15]. These results are in agreement with a recent meta-analysis that demonstrated an association between sporadic MTC susceptibility and S836S polymorphism [16]. Nevertheless, other studies failed to demonstrate any effect of *RET* variants on risk of development or on the natural course of MTC [17–19].

On the other hand, the mechanism by which SNPs modulate the MTC pathogenesis is still open to discussion [12,16]. One of the proposed mechanisms suggests that the S383S neutral variant might be in linkage disequilibrium (LD) with an unknown functional variant, which may modulate the *RET* oncogene expression by affecting the mRNA stability [13,20,21]. Recently, the presence of multiple *RET* risk alleles has been associated with increased estimated risk for MTC development and aggressiveness, indicating that these variants could be acting in an additive manner on disease pathogenesis [22].

The *RET* mutations are also associated with Hirschsprung's disease (HSCR), a developmental disorder of the enteric nervous system, characterized by the absence of ganglion cells in the distal colon resulting in functional obstruction [23]. However, contrasting with the gain-of function effect caused by *RET* mutations in MTC, loss-of-function *RET* mutations account for approximately 50% of familial and 7–35% of sporadic HSCR patients [24,25]. A potential explanation for these distinct effects is that constitutive RET activation is sufficient to induce neoplastic transformation of the C-cells and adrenal chromaffin cells, but not to produce a trophic response in the precursor neurons [26]. Of interest, *RET* polymorphisms and haplotypes have been described as underrepresented in HSCR patients, which could act as low susceptibility loci and modify the phenotype of HSCR [27–29]. Particularly, the haplotype including the uncommon S836S polymorphic allele has shown a low penetrant protective effect against the disease [30,31]. Interestingly, Griseri et al 2007 report a strong LD among S836S and 3’untranslated region (3’UTR) variants (rs76759170, G>A and rs3026785, T>C) [32]. The 3’UTR gene region is emerging as fundamental and effective in regulating gene expression at posttranscriptional levels (pre-mRNA processing, mRNA stability and translational regulation) [33], suggesting that 3'UTR variants might have a functional effect on the RET expression [32].

Here, we evaluated the presence of linkage disequilibrium between S836S and 3’UTR *RET* variants in MTC patients and assessed whether the 3'UTR variants could play a role **in** *RET* expression.

## Material and Methods

### Ethics Statement

The information obtained during the study did not affect the patients’ diagnosis or treatment. The protocol was approved by the Committee on Research Ethics from Hospital de Clínicas de Porto Alegre (project number 12–0225), and all patients and/or their legal representatives provided written informed consent for the study. Clinical investigation was conducted according to the principles expressed in the Declaration of Helsinki.

### Patients

Patients with a diagnosis of MTC attending the Endocrine Division at Hospital de Clínicas de Porto Alegre were invited to participate in the study. Since 1997, our division has been a reference center for *RET* mutation screening in Brazil, and therefore, patients referred to us by other Brazilian centers were also invited to participate.

The patients underwent a complete clinical examination, laboratory tests (levels of basal calcitonin (Until December 2003, Calcitonin IRMA-DSL7700, Diagnostic Systems Laboratories, Inc., Webster, TX, USA, reference range <10 pg/ml and, after January 2004, Immulite 2000, Diagnostic Products Corporation, Los Angeles, CA, USA; reference value (VR) male <12.0 pg/ml and female <6.0 pg/ml)), plasma parathyroid hormone (PTH; Immulite 2000 Intact PTH, Diagnostic Products), urinary fractionated metanephrines (HPLC), and, whenever indicated, diagnostic imaging investigation (cervical ultrasonography, thorax and abdominal computed tomography (CT)). Selected patients were submitted to whole-body metaiodobenzylguanidine scintigraphy to rule out distant metastasis.

In our division, the MTC treatment follows the protocols recommended by the current guidelines [4,5]. Total thyroidectomy was performed in all patients with varying cervical neck dissection procedures. The diagnosis of lymph node metastasis was based on histological examination. Patients with suspicious distant metastasis (i.e. the presence of local metastasis and/or serum calcitonin >150 pg/ml) underwent imaging exams (cervical, thoracic and abdomen CT (or liver magnetic resonance imaging), and bone scintigraphy). Patients with undetectable calcitonin levels were considered free of disease. Tumor staging was performed according to the current International Union against Cancer TNM classification[34].

### Genotyping Assay

*RET* variants L769L (rs1800861, codon 769, exon 13, LeuCTT/LeuCTG), S836S (rs1800862; codon 836, exon 14, SerAGC/SerAGT), S904S (rs1800863, codon 904, exon 15, SerTCC/SerTCG) and 3’UTR (rs3026785, T>C and rs76759170, G>A) were analyzed in DNA extracted from peripheral blood leukocytes by standardized procedures. Genotype analysis was performed using Human Custom TaqMan SNP Genotyping Assays (Applied Biosystems, Foster City, CA, USA), as described previously by [22,35]. Primer and probe sequences used for genotyping the *RET* 3’UTR variants were, rs3026785: 5’-CACGTAAATGCAGAAGTTACTAAGTATTAAGTATTACT-3’ (forward primer), 5’-AGGAACATGATCTGGTTTAATGACCTTT-3’ (reverse primer), VIC-5’-TCTGTCAGTTATTAAAATT-3’, FAM-5’-TGTCAGTTACTAAAATT-3’; rs75759170: 5’-ACACGTAACCTGGCTCTAATTTGG-3’ (forward primer), 5’-CTGCATTTAGTAAGACTATCATTAAGCATATCTGA-3’ (reverse primer), VIC-5’-CACAGTGTATCTGAAAAA-3’, FAM-5’-CACAGTGTATTTGAAAAA-3’.

The reactions were conducted in a 96-well plate, in a total 5 ml reaction volume using 2-10ng genomic DNA, TaqMan Genotyping Master Mix 1X (Applied Biosystems), and Custom TaqMan Genotyping Assay 1X. The plates were then positioned in a real-time PCR thermal cycler (7500 Fast Real PCR System; Applied Biosystems) and heated for 10 min at 95°C, followed by 45 cycles of 95°C for 15s and 62°C for 1min. Fluorescence data files from each plate were analyzed using automated allele calling software (SDS 2.1; Applied Biosystems).

### Haplotype Construction and Linkage Disequilibrium Analysis

The haplotypes were constructed based on the combination of allelic variants and their frequencies were inferred using the phase 2.1 program, which implements a Bayesian statistical method [36]. We also used the phase 2.1 program to compare the distributions of different *RET* haplotypes between MTC patients through permutation analyses of 1000 random replicates [36]. Among all pairs of biallelic loci, we examined widely used measures of linkage disequilibrium (LD), Lewontin’s D’ ǀD’ǀ and r^2^ [37].

### Somatic M918T *RET* Mutation Analysis

Forty seven paraffin-embedded MTC samples were available by sequencing. Samples were sequenced at the Unidade de Análises Moleculares e de Proteínas (Centro de Pesquisa Experimental, HCPA) using ABI 3500 Genetic Analyzer with 50 cm capillaries and POP7 polymer (Applied Biosystems). PCR products were labeled with 5.0 pmol of the primer 5’-AGGGATAGGGCCTGGGCTTC-3’ and 1 μL of BigDye Terminator v3.1 Cycle Sequencing Kit (Applied Biosystems) in a final volume of 10 μL. Labeling reactions were performed in a Veriti® 96-Well Thermal Cycler (Applied Biosystems) with an initial denaturing step of 96°C for 1 min followed by 35 cycles of 96°C for 15 sec, 50°C for 15 sec and 60°C for 4 min. Labeled samples were purified using BigDye XTerminator Purification Kit (Applied Biosystems) and electroinjected in the automatic sequencer.

### Thermodynamic Simulations Methodology

The bioinformatics analyses were realized based on haplotypes inferred by Phase program. Additionally, we also used the haplotype carrying only the S836S variant (TTCGT). No patient harbored only S836S polymorphism was observed in our study population, this variant occurs only in combination with L769L and 3’UTR variants (GTCAC haplotype).

The preliminary analysis was realized in both, wild type (TCCGT_WT haplotype) and polymorphic sequences (GCCGT, TCGGT and GTCAC haplotypes), with the RNAfold algorithm provided by the Vienna Package 2.1.5. This algorithm provides the optimal RNA secondary structure and its associated minimal free energy (MFE) for each input sequence [38].

The Vienna Package [38] program RNAsubopt with default parameters was used to generate suboptimal secondary structures of all haplotypes. Since the number of possible suboptimal structures for each sequence is exceedingly large we randomly selected a subset of those using the -p option. In this analysis we used subsets of 2900 structures for each sequence. The resulting dot-bracket files were evaluated as follows: 1) by counting the number of continuous base-pairs in double helices of at least 6 bp (number of double helices sections—*N*_*DH*_); 2) by calculating the resulting Gibbs free energy (kcal/mol) with RNAeval from the Vienna Package [38].

First we performed these calculations for the wild type sequence and obtained average numbers of base-pairs, total melting index and Gibbs free energies. Then we compared each suboptimal structure of the polymorphic haplotype to those averages and counted how many times they present higher thermodynamic stability (lower Gibbis free energy). This gives us a measure that represents the probability which polymorphic haplotype may result into a more stable structure than the wild-type sequence.

The NCBI Reference Sequence accession number for the *RET* gene was NM_020975.4 (mRNA).

### Immunohistochemistry Analysis

Immunohistochemistry analysis (IHC) was performed on thin sections (3 μm) of previously formalin-fixed and paraffin-embedded tissues. The antibody used was monoclonal rabbit anti-human RET (ab134100; Abcam Inc., Cambridge, MA, USA), Sections representing MTC were submitted to routine immunohistochemical technique, which comprises deparaffination and rehydration, antigenic recovery, inactivation of endogenous peroxidase, and blockage of unspecific reactions. Primary antibodies were incubated overnight at a temperature of 4°C, at dilutions of 1:400 followed by application of streptavidin horseradish peroxidase conjugate (LSAB; DakoCytomation, Via Real Carpinteria, CA, USA), and diaminobenzidine tetrahydrochloride (Kit DAB; DakoCytomation). Absence of the primary antibody was used as a negative control.

### Semi-Quantitative Analysis for the Intensity of Positive Staining in Tissues

The intensity of positive staining of RET was performed by digital image analysis using the Image-Pro Plus 6.0 software (Media Cybernetics, Rockville, MD, USA). Two independent researchers (L.C. and J.O.S) carried out blind analysis of the intensity of brownish-colored immunostaining in pixels in 4 to 12 fields from each slide, according of the tumor size area. The mean number of pixels identified by both researchers in each sample was used. All of the images were taken using the same microscope and camera sets [39].

### Statistical Analysis

Results are expressed as mean ± S.D. or median (IQ 25–75) unless otherwise specified. Hardy–Weinberg equilibrium for each polymorphism was assessed by χ^2^ tests. Baseline characteristics were compared using χ^2^ tests or Fisher’s exact test for qualitative variables. Quantitative variables were compared between groups using Student’s t-test or Mann-Whitney test. The differences in cumulative lymph node and/or distant metastasis between groups were tested by Kaplan–Meier curves; comparisons between curves were performed using the log rank test. The Statistical Package for the Social Sciences 18.0 (SPSS Inc., Chicago, IL, USA) was used, and P<0.05 was considered as statistically significant.

## Results

### *RET* Variants in MTC Patients

The clinical and oncological features of the study subjects are summarized in Table 1. The median age at diagnosis was 48.5±15.5 years and the percentage of women was 57.9%. The ethnic background was 95% Caucasian. The median basal serum calcitonin and CEA levels at diagnosis were 782 (167–2566) pg/ml and 33.6 (7.13–115.4) ng/ml, respectively. The median tumor size was 2.3 (1.5–3.35) cm. Seventy-eight patients (58.3%) presented lymph node and 23.1% presented distant metastasis at diagnosis, respectively. The minor allele frequencies observed were as follows: S836S (5.6%) and 3’UTR *RET* variants rs76759170 (5.6%) and rs3026785 (6.2%) (Table 2).

**Table 1:** The clinical and oncological features of medullary thyroid carcinoma patients.

Total patients (%)	152
Sex female (%)	88 (57.9)
Age at Diagnosis (yr) ^1^	48.5±15.5
Calcitonin (pg/ml) ^2^	782 (167–2566)
CEA (ng/ml) ^2^	33.6 (7.13–115.4)
Tumor Size (cm) ^2^	2.3 (1.5–3.35)
N1 (%)	77 (58.3)
M1 (%)	27 (23.1)

CEA, carcinoembryonic antigen; N1, lymph node metastasis; M1, distant metastasis.

^1^Data expressed as mean±S.D.

^2^Data expressed as median (IQ 25–75).

**Table 2:** *RET* minor allele frequency polymorphisms in medullary thyroid carcinoma patients.

*RET* Polymorphisms	Allele variation	Sporadic MTC (n = 152)
S836S (%)	C>T	5.6
rs76759170 (%)	G>A	5.6
rs3026785 (%)	T>C	6.2

### Haplotype Construction and Linkage Disequilibrium Analysis

We used a Bayesian statistical method to estimate the frequency of different haplotypes produced by the combination of the L769L(rs1800861), S836S(rs1800862), S904S(rs1800863) and 3’UTR(rs3026785 and rs76759170) polymorphisms in MTC patients.

A total of 5 haplotypes were inferred in our study population. The haplotype frequencies were shown in Table 3. Interestingly, the S836S polymorphism occurs only in combination with L769L and 3’UTR variants. We observed a strong LD among S836S and 3’UTR variants: S836S and rs76759170 (|D’| = -1, r^2^ = 1), S836S and rs3026785 (|D’| = -1, r^2^ = 0.967), and rs76759170 and rs3026785 (|D’| = -1, r^2^ = 0.967).

**Table 3:** Haplotypes inferred by the Phase Program.

	Presence/absence of					HaplotypeFrequency^1^
Haplotypes	L769L	S836S	S904S	rs76759170	rs3026785	
TCCGT (WT)						55
GCCGT	X					18
TCGGT			X			21
GCCGC*	X				X	0.3
GTCAC	X	X		X	X	5.6

Haplotype frequencies was calculated by the Phase 2.0 program using permutation test (1000 replications). For simplified representation, the Phase program shows the haplotypes by the presence of the wild-type or risk alleles.

^1^ Frequencies calculated by the Phase 2.0 program based on the number of chromosomes.

*The GCCGC haplotype was present in only two patients, thus was not included in our analyzes.

### *RET* Haplotypes and Disease Presentation

**Fig 1:**
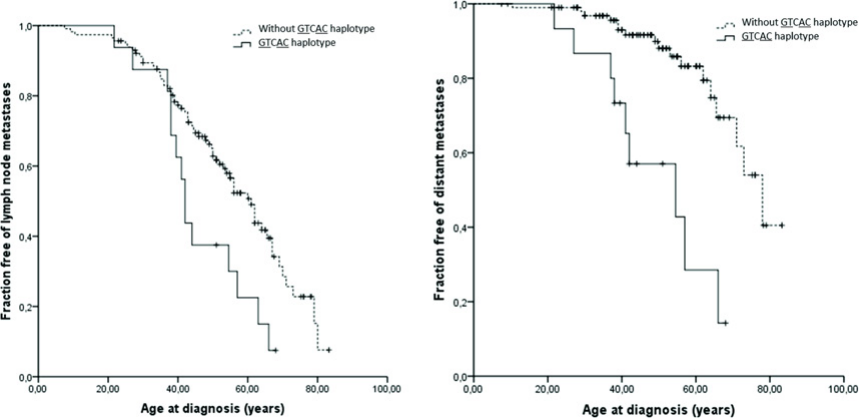
Kaplan–Meier estimates the proportion of sporadic MTC patients with lymph node (A; n = 131; P = 0.011) or distant metastasis (B; n = 116; P<0.001). The log rank test was used to compare curves.

Seventeen patients (11.2%) were heterozygous for S836S/3’UTR polymorphic allele (GTCAC haplotype). Individuals harboring the S836S/3’UTR haplotype (GTCAC) had higher serum calcitonin level (1860(1160–7087) *vs* 579(120–2014) pg/mL, P = 0.014), CEA level (584(145–4353) *vs* 26(7–113) ng/mL, P = 0.007) and larger tumor size (3.3(1.8–4.0) *vs* 2.1(1.4–3.0), P = 0.062). The S836S/3’UTR *RET* haplotype (GTCAC) was associated with higher rates of metastatic disease as compared to patients without GTCAC haplotype (87.5 *vs* 53.9%, P = 0.013 and 60 *vs* 16.8%, P = 0.001) (Table 4). Accordingly, Kaplan–Meier estimates of cumulative lymph node and distant metastasis yielded distinct curves for patients with or without the S836S/3’UTR haplotype (P = 0.011 and P<0.001, respectively; Fig 1), further demonstrating that metastatic disease occurred earlier in those individuals harboring the S836S/3’UTR variants.

**Table 4:** Clinical and oncological features of sporadic medullary thyroid carcinoma patients according to the presence of polymorphic haplotype.

	Without GTCAC	GTCAC	*P*
Total patients (%)*	133 (88.6)	17 (11.3)	
Sex female (%)	79 (59.4)	8 (47.1)	0.332
Age at Diagnosis (yr)	48.6±16	45.6±13	0.441^a^
Calcitonin (pg/ml)^1^	579 (120–2014)	1860 (1160–7087)	0.014^b^^,^^d^
CEA (ng/ml)^2^	26 (7–113)	584 (145–4353)	0.007^b^^,^^d^
Size Tumor (cm)^3^	2.1 (1.4–3.0)	3.3 (1.8–4)	0.062^b^
N1 (%)^4^	62 (53.9)	14 (87.5)	0.013^c^
M1 (%)^5^	17 (16.8)	9 (60)	0.001^c^

PHEO, pheochromocytoma; HPT, hyperparathyroidism; N1, lymph node metastasis; M1, distant metastasis; GTCAC, haplotype harboring L769L, S836S and 3’UTR *RET* variants; Without GTCAC, all other haplotype.

* The total of patients available was 150, the two patients carrying the GCCGC were not included in our analyzes.

^1^ Data evaluated for 67 patients

^2^ Data evaluated for 39 patients

^3^ Data evaluated for 99 patients

^4^ Data evaluated for 131 patients

^5^ Data evaluated for 116 patients

^a^Data expressed as mean±S.D.

^b^Data expressed as median (IQ 25–75).

^c^Variables were compared using the Yates’ X^2^-test or Fisher’s exact test.

^d^Variables were compared using the Mann–Whitney U test.

### Somatic M918T *RET* Mutation

The somatic *RET* M918T mutation has been previously associated with lymph node metastases at diagnosis and could be a confounding factor in our analysis. Thus we also evaluated somatic *RET* mutations in our sample population. Forty-seven paraffin-embedded samples were available. Of them, we failed **to** extract / amplify DNA from 7 even after several repeated attempts. Of the 40 DNA samples available for analysis, 13 (32.5%) samples have somatic M918T mutation. There were no significant difference in the frequency of somatic M918T mutation in samples with or without S836S/3ÚTR variants (10/31 (32.3%) vs. 3/9 (33.3%), P = 1.00; Fisher’s exact test). In addition, no association was observed between the presence of somatic M918T mutation with lymph node or distant metastasis (P = 0.614 and P = 0.628, respectively).

### *In Silico* Analysis of *RET* Haplotypes

Our purpose was to evaluate the effect of *RET* neutral variants on the full *RET* mRNA structure (5.6kb). In order to minimize the experimental limitations, increase the accuracy of computer predictions of RNA secondary structure and perform a more robust analysis, we evaluated the effect of the *RET* haplotypes on the optimal and suboptimal mRNA structures. The bioinformatics analyses used all haplotypes inferred by Phase program (Table 3) and included only synonymous polymorphisms.

**Fig 2:**
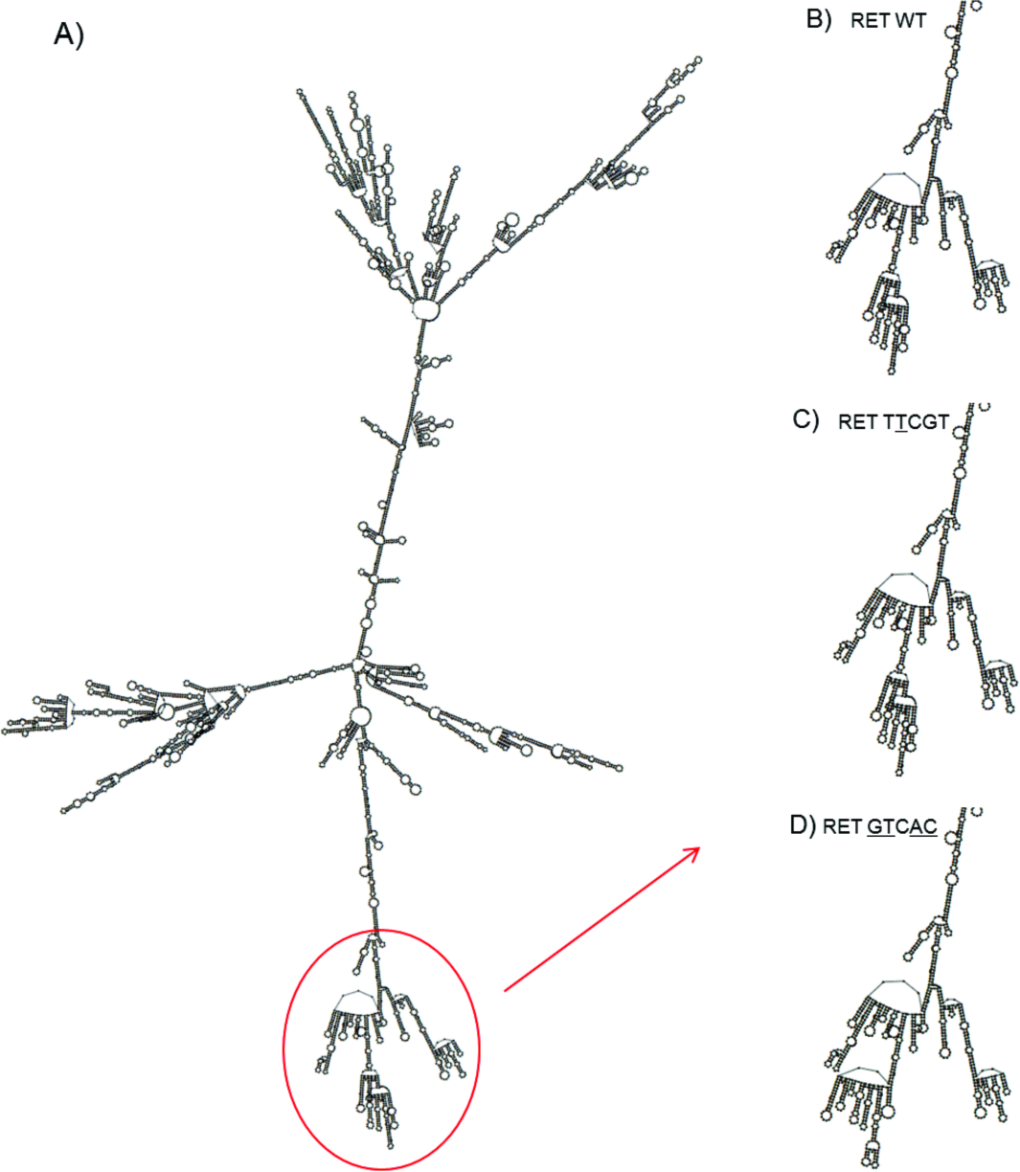
The figure shows the optimal mRNA secondary structure of the RET haplotypes. A) Total mRNA secondary structure of the *RET* wild-type (WT) haplotype. B) Part of mRNA secondary structure of the *RET* WT haplotype. C) Part of mRNA secondary structure of the *RET* S836S haplotype (TTCGT). D) Part of mRNA secondary structure of the *RET* 3’UTR haplotype (GTCAC). Haplotypes generated by RNAfold program (Vienna Package).

As shown in Fig 2, RNA folding analyses have demonstrated different RNA secondary structure predictions for WT (TCCGT), S836S (TTCGT) and S836S/3’UTR (GTCAC) haplotypes. We can observe that S836S/3’UTR (GTCAC) haplotype presented a structural modification not observed in WT (TCCGT) or S836S (TTCGT) haplotypes.

**Fig 3:**
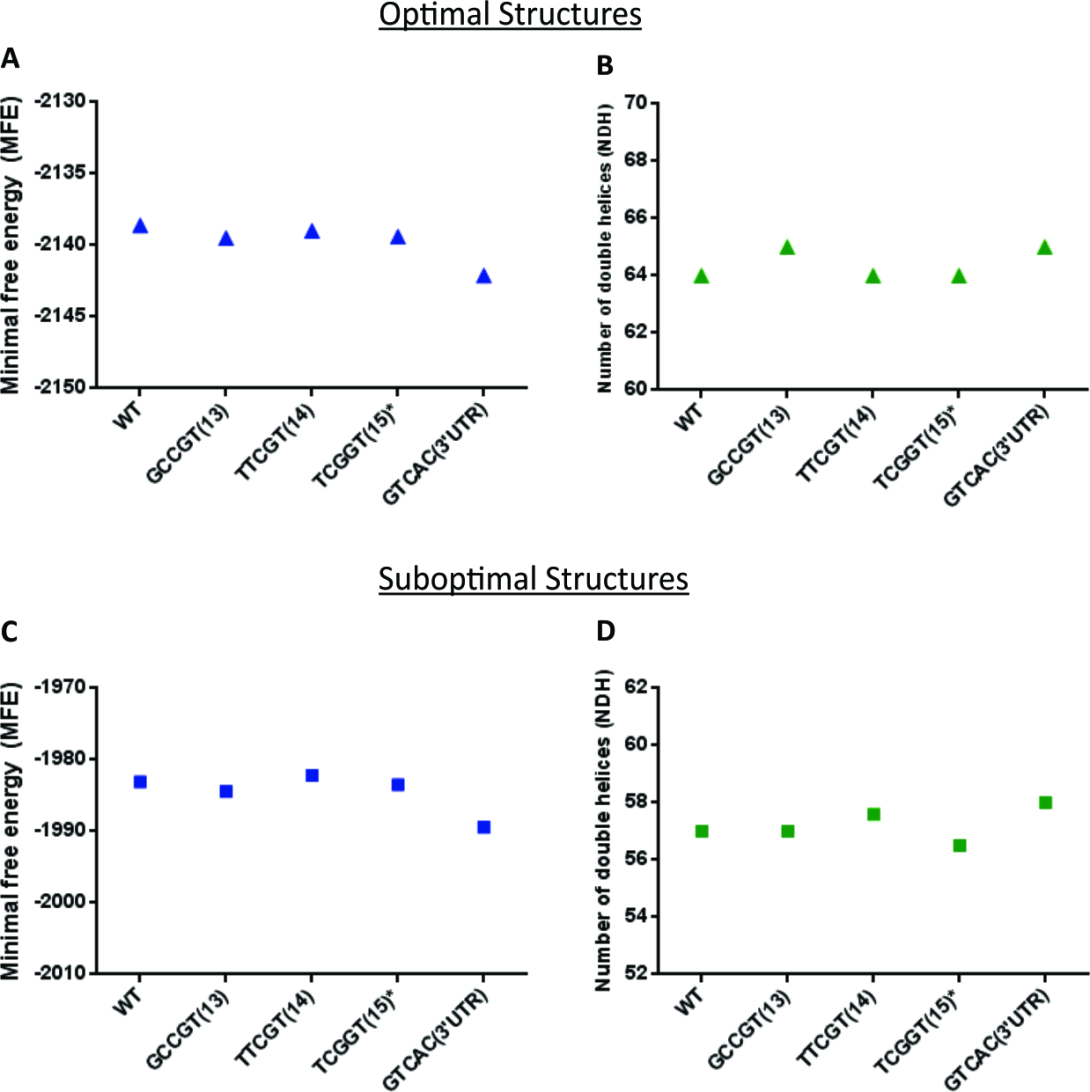
The minimal free energy (MFE, kcal/mol) and number of double helices (*N*_*DH*_) were available in both, optimal and suboptimal structures. For suboptimal structures MFE and NDH are averages over 2900 samples. The variant fragment carrying the S836S and 3’UTR variants (GTCAC haplotype) presented greater *N*_*DH*_ (B,D) and lower levels of MFE (A,C) when compared to wild-type haplotype (WT, TCCGT), this fact happens in both, optimal and suboptimal structures. *These analysis included only synonymous polymorphisms.

Concerning the *N*_*DH*_ analyses, the S836S/3’UTR (GTCAC) haplotype presented greater *N*_*DH*_ in both, optimal and suboptimal structures when compared with the WT haplotype (65 *vs* 64, Fig 3B and 58 *vs* 57, Fig 3D, respectively). In agreement, the sequence carrying S836S/3’UTR (GTCAC) haplotype presented lower levels of MFE when compared to the WT sequence (optimal, -2142.1 *vs* -2138.6, Fig 3A; suboptimal, -1989.4 *vs* -1983.1, Fig 3C). Thus, the structure carrying the 3’UTR variants provides the most thermodynamically stable mRNA secondary structure when compared to the other.

### Immunohistochemistry Analysis

Next, we decided to look whether the presence of the S836S/3’UTR (GTCAC) haplotype was associated with increased expression of RET protein in paraffin-embedded samples of patients with or without this haplotype. To minimize confounding factors, we selected only samples negative for somatic M918T mutation, since studies have demonstrated association between the presence of this mutation and stronger RET immunostaining [40].

**Fig 4:**
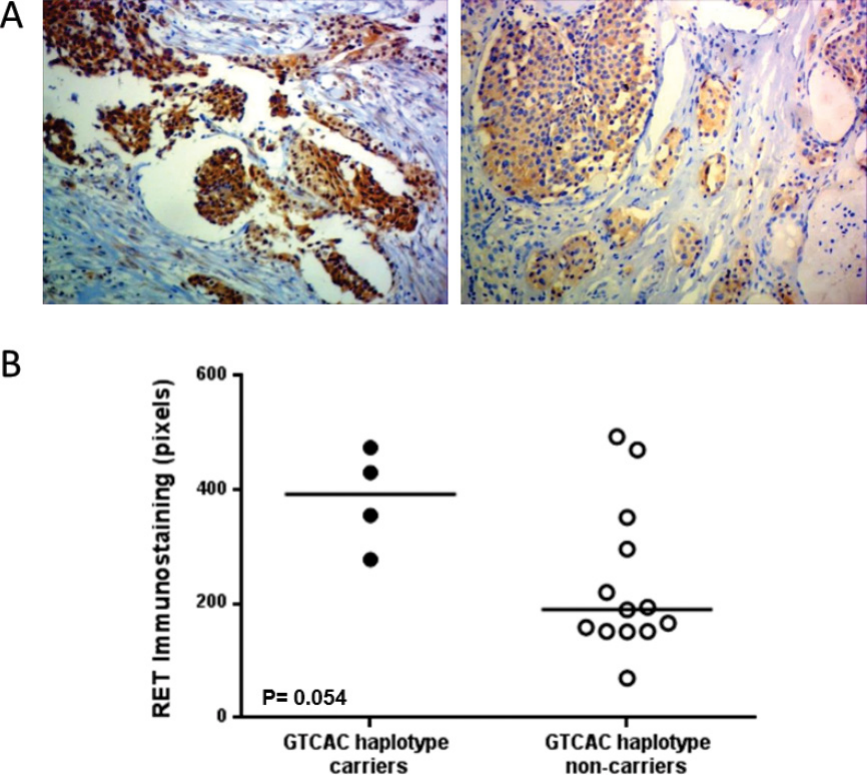
Immunostaining of the RET proto-oncogene in GTCAC haplotype carriers & non-carriers. A) Two representative slices of RET Immunostaining in a sample carrier S836S/3’UTR (GTCAC haplotype) (left) and non-carrier of this haplotype (right). B) Intensity of RET Immunostaining in samples with or without S836S/3’UTR (GTCAC haplotype), P = 0.054.

Four samples carrying S836S/3’UTR (GTCAC) and 13 samples without the variants were available for analysis. Immunostaining for RET were detected in 100% of the samples analyzed. The overall median of the RET immunoreactivity observed was 220.2 (155–393) pixels. Of interest, all MTC samples carrying S836S/3’UTR (GTCAC) haplotype uniformly display a very strong intensity of RET immunostaining (Fig 4A). Remarkably, despite the relative small number of samples, we observed a strong trend towards a significant statistical difference in the RET immunostaining between samples carrying S836S/3’UTR (GTCAC) haplotype and those without these variants (392.7 (297–463) vs. 189.5 (152–324), P = 0.054; Fig 4B).

## Discussion

In the present study, we have demonstrated that the neutral *RET* S836S polymorphism is in linkage disequilibrium with the *RET* 3’UTR variants (rs3026785 and rs76759170) in patients with sporadic MTC. Interestingly, the individuals harboring these variants presented more aggressive disease and metastasis at younger age. Furthermore, the haplotype carrying the S836S/3’UTR *RET* variants presented a greater *N*_*DH*_ and lower levels of MFE as compared to the wild-type haplotype, which may have functional consequences on the rate of mRNA degradation. Interestingly, MTC samples carrying S836S/3’UTR (GTCAC) haplotype displayed stronger RET immunostaining than those without these variants, although the difference did not reach statistical significance.

Based on the overrepresentation of *RET* polymorphisms in individuals with MTC, a role for these genetic variants in the MTC pathogenesis has been suggested. However, the molecular mechanism by which *RET* S836S polymorphism affects the development or clinical course of MTC is still not properly understood. It has been suggested that polymorphisms could influence the *RET* mRNA levels. However, quantitative studies in MTC tumor samples show no difference in *RET* mRNA levels in patients with or without the S836S polymorphisms [41]. Another hypothesis suggests that bases exchange in the DNA molecule could create a new alternative splicing site, leading to the synthesis of a truncated protein, erroneous ligand binding, microRNA (miRNA) binding, change of structure and mRNA stability as well as a number of copies [42]. However, the S836S polymorphic variant does not affect DNA–protein binding or RNA splicing and editing of the *RET* gene [43]. An alternative way to explain the role of *RET* S836S polymorphisms on MTC proposes that this neutral variant can be in LD with an unknown functional variant [44].

Of interest, a strong LD between S836S polymorphism and 3’UTR variants has been reported in HSCR disease [32]. The 3’UTR gene region is emerging as fundamental and effective in regulating gene expression at posttranscriptional levels (pre-mRNA processing, mRNA stability and translational regulation) [33]. Several sequence elements that may be involved in mRNA regulation exist in the 3′UTR region, including regions rich in adenine and uridine elements—AU-rich elements (ARE). The ARE mRNAs are regulated by RNA-binding proteins that can selectively bind to the ARE and promote their mRNA stability or degradation [45]. Elegant studies performed by Griseri et al [31,32] demonstrated that the rs3026785 is located next to the AUUUA sequence and showed that a single nucleotide substitution may influence the secondary structure of *RET* mRNA decreasing *RET* mRNA degradation in human neuroblastoma cells, leading to increases of transcription product and, probably, in the amount of total *RET* protein at the cell membrane [31,32].

The S836S polymorphism is associated with increased RET expression in both diseases. Thus, interestingly, in HSCR, the S836S variant is associated with a protective effect since loss-of-function RET is causative of the disease [31,32]. We have previously shown that the *RET* S836S polymorphism is overrepresented in sporadic MTC patients [22]. Here, we show that the *RET* S836S polymorphism is in linkage disequilibrium with *RET* 3’UTR variants in MTC patients. Of interest, patients harboring these variants present higher levels of serum calcitonin and CEA, larger tumor size, and higher rates of metastatic disease as compared to those harboring other haplotypes. The somatic *RET* M918T mutation has been associated with advanced disease at diagnosis [9,46] and could be a confounding factor in our analysis. However, we found no significant difference in the frequency of somatic M918T mutation in samples from patients with or without S836S/3UTR variants.

To further explore the modulating effect of *RET* S836S/3’UTR polymorphisms, we thought to evaluate the effects of the 3’UTR variants on the mRNA structure. Thermodynamics-based computational methods for prediction of RNA secondary structure are widely used to advance our understanding of the regulatory roles of cellular RNA [47,48]. These methods use MFE principles to find the RNA secondary structure from the set of all possible structures for a given RNA sequence. In this study, *in silico* analysis showed that the S836S/3’UTR haplotype (GTCAC) could influence the stability of the *RET* mRNA. We observed that this haplotype presents the highest *N*_*DH*_ and lower levels of MFE, suggesting that *RET* mRNA carrying the S836S/3’UTR haplotype provides the most thermodynamically stable mRNA structure, when compared to the others. Thus, our data suggest that the S836S/3’UTR haplotype may affect the secondary structure of *RET* mRNA, supporting the hypothesis of a functional involvement of the 3’UTR variant allele in *RET* mRNA stability. Based on the results presented here, we speculated that S836S variant promotes an increased activity of RET by increasing the mRNA survival, contributing for gain-of-function RET that is causative of MTC. Interestingly, immunohistochemical analysis shows that samples carrying S836S/3’UTR (GTCAC) haplotype displayed stronger RET staining intensity than those without these variants (Fig 4). Although the difference did not reach statistical significance, these observations may indicate an effect of the polymorphic haplotype on RET expression.

Potential miRNAs recognition site are identified in 3' UTR regions, thus, it is reasonable to speculate that the 3’UTR polymorphism could be involved in a ligation site for a given miRNA, interfering the *RET* expression. Therefore, we used the TargetScan and miRDB softwares to look for potential miRNA targets in the 3’UTR of *RET* gene. We can observe that the *RET* 3’UTR variants (rs3026758 and rs76759170) are situated near to several miRNA-binding sites, but none of them is situated in the miRNA seed region. The miRNA-binding sites for the hsa-miR-27a, hsa-miR-27b, hsa-miR-128a, hsa-miR-216a-3p and hsa-miR-3681-3p are situated near to rs76759170, although this variant is not in the miRNA seed region. Similar results were found to the hsa-miR-590-3p and the rs3026785. Additional studies are warranted to further explore this issue.

## Conclusion

Our results demonstrate linkage disequilibrium between *RET* S836S and 3’UTR genetic variants. The patients harboring S836S/3’UTR haplotype presents more aggressive disease as compared to those harboring the other haplotypes. Furthermore, the *RET* mRNA sequence carrying the S836S/3’UTR haplotype present higher structural and thermodynamic stability, supporting the hypothesis of a functional involvement of the 3’UTR variant allele in the posttranscriptional control of a *RET* transcripts
